# Information needs and experiences from pregnancies complicated by hypertensive disorders: a qualitative analysis of narrative responses

**DOI:** 10.1186/s12884-021-04219-0

**Published:** 2021-11-02

**Authors:** Raj Shree, Kendra Hatfield-Timajchy, Alina Brewer, Eleni Tsigas, Marianne Vidler

**Affiliations:** 1 Division of Maternal Fetal Medicine, Department of Obstetrics & Gynecology, University of Washington, 1959 NE Pacific Street, Box 356460, Seattle, WA 98195 USA; 2 Health Scientist, Division of Reproductive Health, Centers for Disease Control and Prevention, Atlanta, GA USA; 3 Preeclampsia Foundation, Predictive Laboratories, Inc., Melbourne, FL USA; 4 Preeclampsia Foundation, Melbourne, FL USA; 5 Department of Obstetrics & Gynecology, British Columbia Children’s Hospital Research Institute, University of British Columbia, Vancouver, BC Canada

**Keywords:** Preeclampsia, Hypertensive disorders of pregnancy, Patient experience, Qualitative research

## Abstract

**Background:**

Incorporation of the patient voice is urgently needed in a broad array of health care settings, but it is particularly lacking in the obstetrical literature. Systematically derived information about patients’ experience with hypertensive disorders of pregnancy (HDP), most notably preeclampsia, is necessary to improve patient-provider communication and ultimately inform patient-centered care and research.

We sought to examine the information needs and experiences of individuals with pregnancies complicated by hypertensive disorders.

**Methods:**

We conducted a qualitative content analysis of narrative-responses to an open-ended question from the Preeclampsia Registry (TPR), an online registry hosted by the Preeclampsia Foundation. Individuals were invited to enroll in TPR via social media, web searches, and newsletters. We restricted our analysis to participants who self-reported a history of HDP and responded to the open-ended question, “*Is there any information that you could have had at the time of this pregnancy that would have been helpful?*”. Available responses from July 2013 to March 2017 were included. Narrative responses were coded, reconciled, and thematically analyzed by multiple coders using an inductive approach. Our main outcome measures included participants’ expressed needs and additional concerns with respect to their HDP pregnancy.

**Results:**

Of 3202 enrolled participants, 1850 completed the survey and self-reported having at least one pregnancy complicated by HDP, of which 895 (48.4%) responded to the open-ended question. Participants delivered in the United States (83%) and 27 other countries. Compared to non-responders, responders reported more severe HDP phenotypes and adverse offspring outcomes. We identified three principal themes from responses: patient-identified needs, management and counseling, and potential action. Responses revealed that participants’ baseline understanding of HDP, including symptoms, management, therapeutic strategies, and postpartum complications, was demonstrably lacking. Responders strongly desired improved counseling so that both they and their providers could collaboratively diagnose, appropriately manage, and robustly and continuously communicate to facilitate a partnership to address any HDP complications.

**Conclusions:**

Participants’ responses regarding their HDP experience provide indispensable insight into the patient’s perspectives. Our study suggests that improved education regarding possible HDP complications and transparency about the consideration of HDP and its associated outcomes during an evaluation are needed, and efforts to implement these strategies should be sought.

**Trial registration:**

The Preeclampsia Registry: NCT02020174

## Background

Hypertensive disorders of pregnancy (HDP) complicate roughly 10% of pregnancies worldwide [1] and approximately 10–15% of maternal mortality can be attributed to preeclampsia or eclampsia, common HDP phenotypes [2–4]. The incidence of preeclampsia is rising in the United States [5–8] and may also be rising worldwide [9, 10]. Better understanding maternal morbidity related to HDP is important given the immediate and long-term impact on maternal and child well-being [11–14] along with the associated costs [15, 16]. Important stakeholders are the women themselves and research into the voice of women with HDP pregnancies is lacking [17]. Knowledge of patients’ perceived experiences may augment our understanding, and thus connection and communications, with patients confronting this significant and potentially traumatic life event. Patient engagement and involvement also may improve outcomes [18], further supporting efforts to systematically understand the patient perspective.

The Preeclampsia Registry (TPR), is an online patient-facing registry launched by The Preeclampsia Foundation in 2013 [19]. It is a “living database” of patient-reported clinical data along with open-ended questions that capture the patient perspective through narrative responses. Individuals that have had an HDP pregnancy, their family members, and controls begin participation in TPR by providing online consent and responding to a series of questions. Among participants of TPR who self-identified as having an HDP pregnancy, we analyzed narrative responses to an optional open-ended question regarding what information they would have liked to have at the time of their HDP pregnancy. Such qualitative research can provide novel insights into the patient experience, catalyze practice changes, and is hypothesis-generating.

## Methods

### Setting and participant selection

We performed content analysis of responses to an open-ended question submitted by TPR enrollees between July 2013 and March 2017. Individuals are recruited to TPR through social media, web searches, and emailed invitations, and complete an online questionnaire self-reporting their pregnancy details and outcomes. Individuals loop through questions for each pregnancy, after which the open-ended question, “*Is there any information that you could have had at the time of this pregnancy that would have been helpful?*” appears. Personalized narrative responses entered into the free-text box were coded, reconciled, and thematically analyzed using an inductive approach.

“Participants” were those who self-reported having an HDP pregnancy and defined our final study population. If an individual had multiple HDP pregnancies, we included them once and their clinical data from the first HDP pregnancy for demographic analysis. Participants with a narrative response to the open-ended question were designated as “responders” and those who did not enter a response or entered “N/A” as “non-responders”. We cannot quantify non-participation as it is unknown how many visited the TPR website but chose not to enroll.

Participants that had an HDP pregnancy selected one or more diagnoses: worsening chronic hypertension, preeclampsia, eclampsia, or HELLP (hemolysis, elevated liver enzymes, low platelet count) syndrome. Gestational hypertension was not included at the time the questionnaire was administered. Human subject protection was approved by Advarra Institutional Review Board and all research was performed in accordance with the Declaration of Helsinki. All participants provided informed consent through an online process.

### Data collection

Demographic information and HDP-related clinical data is collected about each pregnancy. Participants self-report race and ethnicity. For participants with multiple pregnancies, only the open-ended responses from the HDP pregnancies were included. For responders with multiple HDP pregnancies, responses were individually coded and then combined to analyze the data by responder. Offspring characteristics from the first HDP pregnancy were used for demographic analysis (Table 1).

**Table 1:** Participant demographics by first pregnancy complicated by hypertensive disorder of pregnancy

Maternal Characteristic	Responders (***n*** = 895)	Non-Responders (***n*** = 955)	***p-***value
Hispanic	5 (0.6)	10 (1.1)	0.24
Race			
White	768 (85.8)	829 (86.8)	0.53
Black	9 (1.0)	16 (1.7)	0.21
Other^a^	113 (12.6)	100 (10.5)	0.15
More than one pregnancy affected by HDP	221 (23.2)	235 (26.3)	0.12
**Pregnancy Characteristic**	**Responders** **(n = 895)**	**Non-Responders** **(n = 955)**	***p-*value**
Years between pregnancy and completion of survey (or added pregnancy), years (mean ± SD)	5.5 (± 7.1)	4.2 (± 5.8)	< 0.001
Maternal age at delivery, years (mean ± SD)	29.5 (± 5.2)	29.0 (± 5.0)	0.07
Delivery occurred in the US	763 (85.6)	777 (83.3)	0.17
Type of prenatal provider			
Obstetrician	661 (73.9)	684 (71.2)	0.75
Midwife	115 (12.8)	132 (13.8)	
Family practice	53 (5.9)	68 (7.1)	
Other	66 (7.4)	71 (7.4)	
Parity			
0	824 (92.1)	860 (90.0)	0.43
1	52 (5.8)	69 (7.2)	
2	11 (1.2)	20 (2.1)	
≥ 3	8 (0.9)	6 (0.6)	
Multiple gestation	30 (3.3)	39 (4.1)	0.40
Smoking	62 (6.9)	69 (7.3)	0.80
Type of HDP			
Preeclampsia	646 (72.2)	732 (76.7)	0.03
Eclampsia	66 (7.4)	47 (4.9)	0.03
HELLP syndrome	381 (42.6)	372 (38.9)	0.11
Exacerbation of CHTN	59 (6.6)	65 (6.8)	0.85
Unsure of or did not receive a diagnosis	66 (7.4)	68 (7.1)	0.83
Clinical Abnormalities			
Elevated liver function tests	513 (60.1)	508 (55.9)	0.02
Kidney problems	206 (24.2)	159 (17.5)	< 0.001
Low platelets	415 (48.7)	409 (45.1)	0.03
Fluid in the lungs	94 (11.0)	74 (8.2)	0.02
Seizure	57 (6.7)	29 (3.2)	0.003
Increased protein	662 (77.6)	726 (80.1)	0.23
Maximum SBP, mmHg (mean ± SD)^b^	184 (± 26.7)	185 (± 26.9)	0.51
Maximum DBP, mmHg (mean ± SD)^c^	112 (± 18.1)	113 (± 18.8)	0.32
**Offspring Characteristics**^**d**^	**Responder** **(*n*** **= 929)**	**Non-Responder** **(*n*** **= 1009)**	***p-*value**
Gestational age at delivery, weeks (median, range)	34 (20–44)	34 (20–44)	0.83
Cesarean delivery	629 (68.7)	676 (68.2)	0.80
Birth weight, grams (mean ± SD)^e^	2051 (± 987)	2065 (± 975)	0.75
Female sex	472 (51.2)	469 (47.3)	0.23
Offspring outcome			
Live birth with living child	817 (88.2)	909 (91.2)	0.02
Live birth with subsequent infant death	59 (6.4)	59 (5.9)	
Stillbirth	50 (5.4)	29 (2.9)	

HDP, hypertensive disorder of pregnancy; US, United States; HELLP, hemolysis elevated liver enzymes low platelets; CHTN, chronic hypertension

^a^ Includes the following possible responses: Asian, Asian Indian, American Indian or Alaskan Native, Native Hawaiian or Pacific Islander, Other, I don’t know, I’d rather not say

^b^ SBP, systolic blood pressure (included if SBP 60–270 mmHg)

^c^ DBP, diastolic blood pressure (included if DBP 40–180 mmHg)

^d^ Includes data for more than one offspring in multiple gestation pregnancies

^e^ Included if reported birth weight 150–8165 g

Participants could select more than one HDP diagnosis and all were included for analysis. Participants could also indicate that they never received a diagnosis or were unsure of their ultimate HDP diagnosis. Additionally, some participants did not select an HDP type, nor did they indicate being uncertain of their HDP diagnosis. These two groups were combined into participants who were unsure of or did not receive an official diagnosis (*n* = 134).

Reported maximum systolic and diastolic blood pressures (SBP and DBP) were restricted to 60–270 mmHg and 40–180 mmHg, respectively, excluding 49 values. Offspring birth weight was restricted to 150–8165 g, excluding 2 values. In 14 cases of multiple gestations, participants reported a fetal loss due to miscarriage, thus these were designated as singleton gestations.

### Data analysis

Using conventional content analysis, we derived codes for classification from the data. We reviewed a sampling of the responses to get an overall impression to generate initial codes, with additional modifications to the codebook with ongoing review of responses. Three coders (RS, KHT, MV) individually coded 85% of responses, discussed, and reconciled the codes until the codebook was finalized. The remainder of the data was double coded (KHT, MV) using the final codebook. For the entire dataset, consensus regarding the code meaning and application for each response was reached amongst the three coders. Once coding was complete, RS, KHT, and MV examined the data to identify overarching themes and additional sub-themes as applicable. RS, KHT, and MV identify as female.

**Fig. 1:**
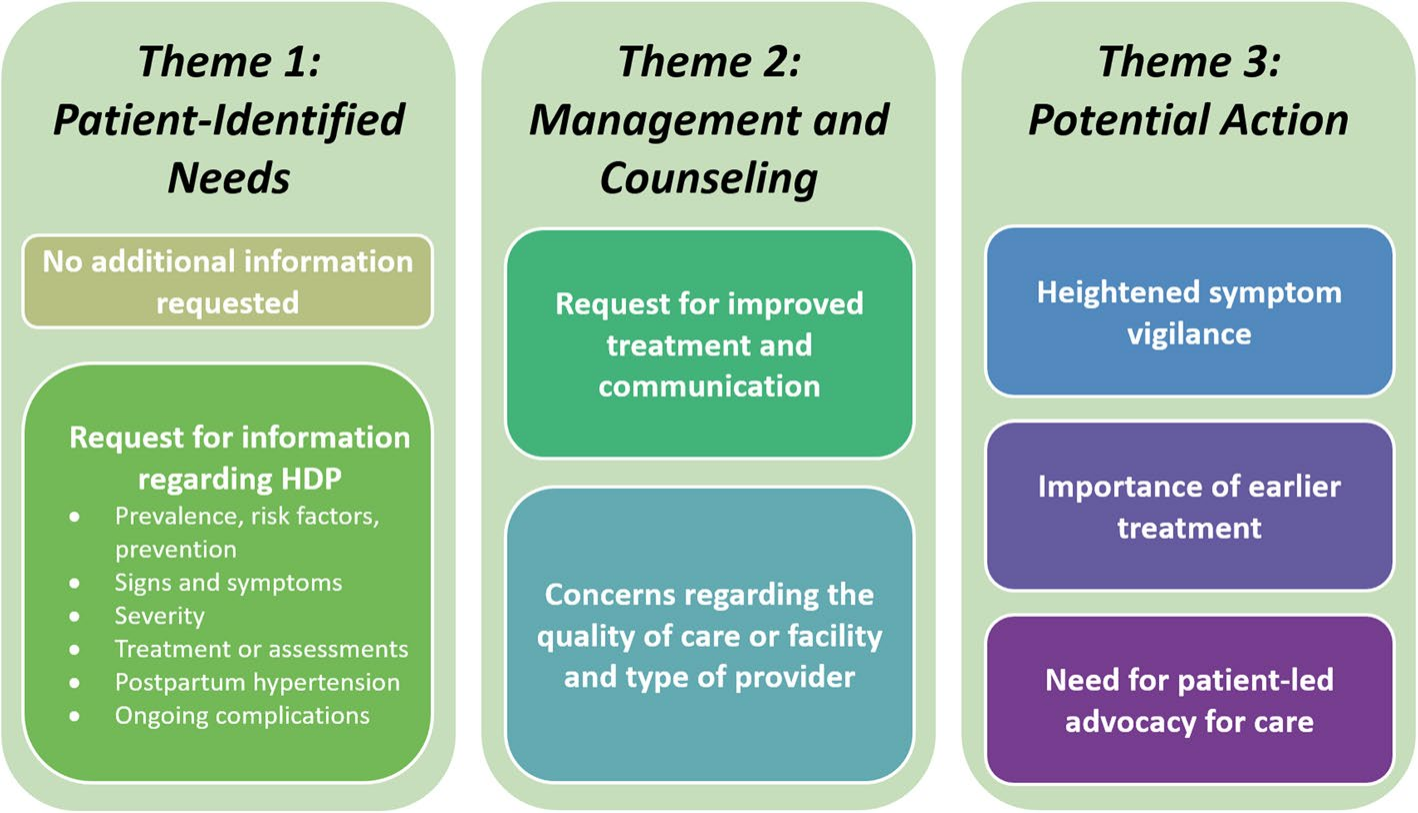
Flowchart of the identified themes and subthemes in our participant cohort

We identified three overarching themes, and additional subthemes were generated based on the responses (Fig. 1). Once responses were grouped by overarching theme and subtheme, they were divided among the coders, summaries were individually written (RS, KHT, MV), and then reviewed until consensus was reached. Through these summaries, we collated the main findings by subtheme under each overarching theme, putting them in prose format, including supporting quotes. Quotes included in each section are from distinct participants.

Participant demographics were analyzed using χ2, Fisher’s exact, *t*-test, or Kruskal-Wallis tests as appropriate. A *p* value of < 0.05 was considered statistically significant. NVivo (version 11, QSR International) was used to index and analyze the narrative data. Demographic data was analyzed using Stata IC 13 (StataCorp, College Station, TX, USA).

## Results

**Fig. 2:**
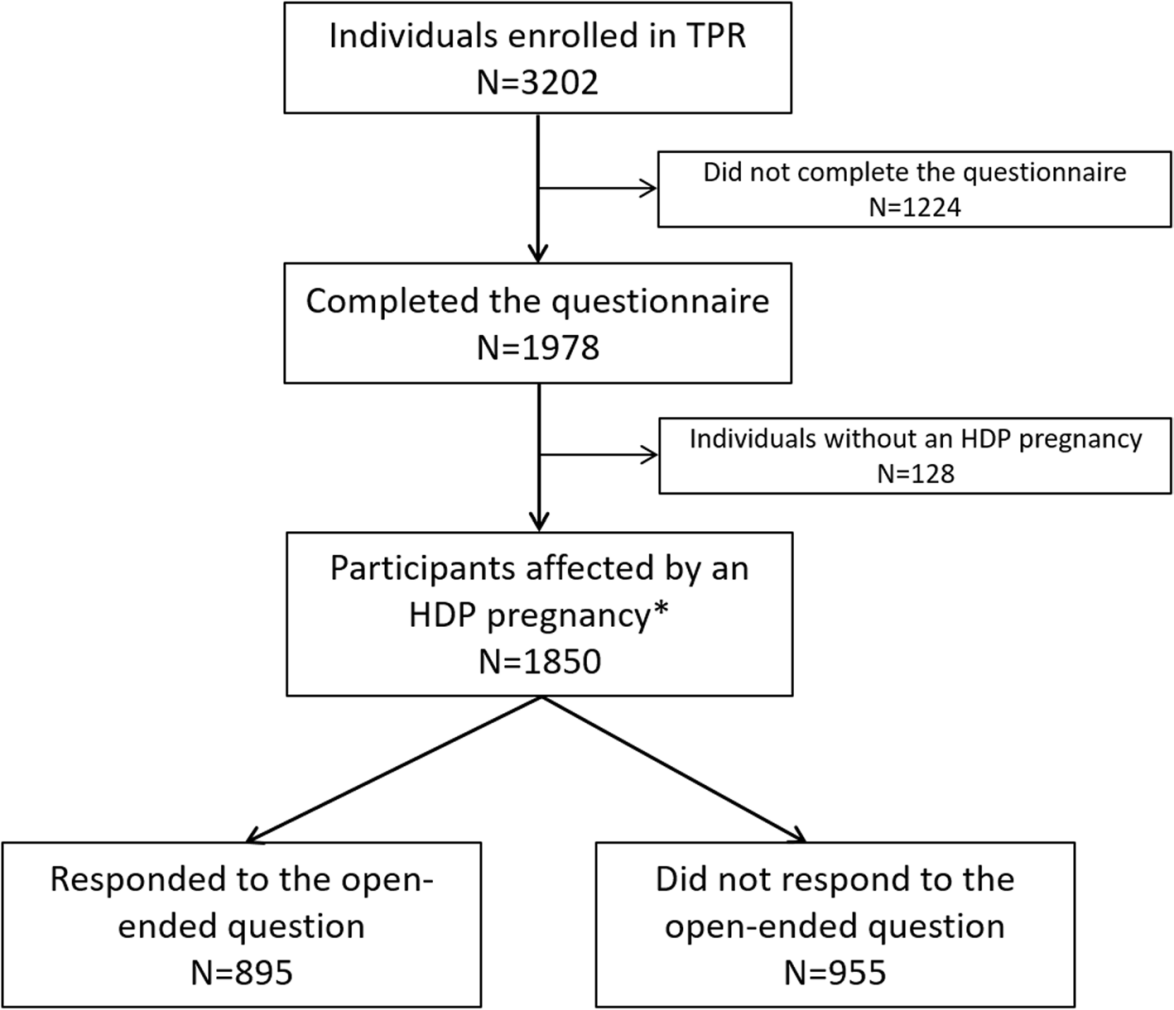
Flowchart demonstrating the study population. TPR, The Preeclampsia Registry; HDP, hypertensive disorders of pregnancy. *, Includes preeclampsia, eclampsia, HELLP syndrome, and chronic hypertension. Also included are participants who indicated having an HDP pregnancy but were unsure of their ultimate diagnosis or did not indicate a specific HDP diagnosis

From July 2013 to March 2017, TPR enrolled 3202 participants. The questionnaire was completed by 1978 (61.8%) participants and 1850 (57.8%) self-reported at least one HDP pregnancy. Of these, 895 participants entered a response to the open-ended question (“responders”), yielding a response rate of 48.4%, while 955 (51.6%) did not enter a response or entered “N/A” (“non-responders”) (Fig. 2).

Most participants self-identified as white and delivered in the US. Participants reported a high rate of recurrent HDP with greater than 20% having more than one HDP pregnancy. There were no significant differences between responders and non-responders with respect to maternal age at delivery, parity, frequency of multiple gestation, smoking during pregnancy, gestational age at delivery, and maximum reported SBP and DBP. There was a longer interval from affected pregnancy to completion of the questionnaire among responders. Responders reported a higher frequency of eclampsia, lab abnormalities, stillbirth, and live birth with subsequent infant death compared to non-responders (Table 1).

We included the number of responders for each subtheme given the large sample size. Describing results using only descriptive terms would be inadequate given the numeric range of possible responses. The terms “few”, “some”, “many”, and “most” corresponds to approximately 5, 15, 30, and 50% of respondents, respectively.

### Participant identified needs

#### Additional information not deemed to be helpful

Of the 895 responders, 195 were satisfied with the information received during pregnancy. An additional 15 made reference to uncertainty about whether information could have changed the outcome, certainty about how nothing could have changed the outcome, or that they accepted, resolute or resigned, the outcome they experienced.

*“I don’t think anything I could’ve done differently would’ve made a difference. Would it?”*

#### Additional information requested

Close to a quarter of the responders (209/895) reported that they were completely unaware of preeclampsia (or other HDPs). For the most part, these responders stated they “*knew nothing about preeclampsia at the time*”. Many of these responders (*n* = 90) were unaware of HELLP in particular prior to symptoms or diagnosis: “*I had never heard of HELLP [and] knew nothing about it or the symptoms.*” Few responders (*n* = 15) raised concerns about the insufficient supply or quality of literature available.

“*I … wish there was a pamphlet*”, “*I don’t recall getting any literature*”, “[*I wish there was] more information on HELLP in the pregnancy books*”.

##### Prevalence, risk factors, and prevention of HDP

Responders conveyed a need for more information on the prevalence, risk factors, and prevention of HDPs (86/895). The most common risk factor raised was the role of previous preeclampsia (*n* = 26) and there was uncertainty regarding risks in subsequent pregnancies: “*It would have been nice to know that having preeclampsia increased my chances of having it again.*” Responders (*n* = 19) expressed a need to be more informed of the role of family history and some (*n* = 11) wanted to know more about the role of pre-existing conditions, including polycystic ovary syndrome, antiphospholipid antibody syndrome, systemic lupus erythematosus, cardiac diseases, and autoimmune, thyroid, and kidney disease: “*Knowing that autoimmune disorders are more likely to trigger HELLP would have at least made me more conscientious.”* Other responders (*n* = 13) wanted to better understand how to prevent HDPs including the role of mitigating stress, diet modification, and aspirin therapy. Some responders (n = 11) were disappointed that their providers did not inform them how to prevent HDP.

##### Signs and symptoms

About one quarter of the responders (231/895) wanted more information on signs and symptoms. More than half of these (*n* = 135) wanted information on the symptoms or stated they did not know the symptoms prior to onset. Few (*n* = 8) claimed to be well-informed on the symptoms of the HDPs.

“*If I knew that headaches combined with swelling were a sign of something more dangerous, I probably would have called the doctor sooner..*.”

Despite being unaware of the symptoms of HDP, many mentioned experiencing symptoms known to be associated with HDP: epigastric pain (*n* = 35), headache (*n* = 20), visual disturbances (*n* = 16), swelling/weight gain (*n* = 53), and shortness of breath (n = 13); indicating a good knowledge of the condition post-hoc. Others were aware of the clinical signs, either because they mentioned experiencing them, or remarked about how they had not; including, proteinuria (*n* = 12), anuria (n = 2), and hypertension (*n* = 28). There was difficulty among these responders (*n* = 45) about how to differentiate HDP symptoms from those of normal pregnancy: “*[I] had all of them except for abdominal pain but passed them off as ordinary pregnancy symptoms*”. A few confused the symptoms of preeclampsia with the onset of labor (*n* = 5). One responder asked about fetal health and whether kick counts could have helped prevent stillbirth.

##### Severity of HDPs

Of 65 responders (out of 895) who desired more information about the severity of HDP, 44 reported being unaware of how serious HDP could be, and 18 expressed regret about not having done more because they did not understand the seriousness of their condition: “*I went home and had a seizure a little over 48 hours later. I had no idea that was even a risk/possibility … I also did not understand the seriousness of the diagnosis until several months later.*” Ten responders remarked how quickly their illness progressed and some (*n* = 8) attributed their rapid decline to being unaware or uninformed about how seriously to take their symptoms. “*I wish I would have known just how quickly the disease can progress - I went downhill FAST - in just a matter of hours*.” “*I had no idea that I was as sick as I was. I thought all my symptoms were normal pregnancy symptoms. I [could] hardly see by the time I was diagnosed and I thought [it] was normal because I was told it was*.”

##### Treatments or assessments of HDP

Of 48 responders (out of 895) who discussed a need for further information on treatments or clinical assessments, 28 wondered whether they should have pursued additional management or therapies. They expressed interest in understanding what blood pressure and proteinuria values are abnormal and learning about the role of blood thinners, antihypertensive agents, and dietary supplements. Responders (*n* = 26) raised broad interest in the role that nutrition and diet may play in preventing or managing their condition. Seven responders were specifically concerned about magnesium therapy, its side-effects, and how it may affect labor and their neonate.

“.*.. it would have been helpful to know that a person’s BP could spike and that [a] high BP reading should be taken seriously even if it is followed by a normal reading. Had I known this I would have pressed my doctor about the issue and maybe could have saved myself from having eclampsia.*”

“*Whether limiting salt [ … ] could have been helpful in stabilizing my blood pressure prior to emergency delivery.”*

*“Would have been nice to know what happens when a person overdoses on magnesium sulfate [ … ] I couldn’t move my muscles and any time I tried to fall asleep, I’d stop breathing.”*

##### Postpartum hypertension

A number (35/895) of responders expressed a desire for more information regarding HDP postpartum. Most of these (*n* = 23) were concerned about their ignorance of postpartum HDP, that their disease status could worsen, and that symptoms could persist postpartum.

*“I wish I had been kept in the hospital longer for observation... My blood pressure was not stabilized … I went home and had a seizure a little over 48 hours later. I had no idea that was even a risk/possibility. I did not even know postpartum preeclampsia existed, until I had it.”*

*“I didn't know how severe of an illness this is until I lost my vision after delivery. I had no idea that I was still at risk post-delivery.”*

These responders (*n* = 7) also expressed concern about inappropriate or inadequate counseling during the postpartum period.

*“My mom and my sister are both delivery nurses and they were shocked that I left the hospital with a bottle of Labetalol and no follow up appointments, no doctor overseeing my recovery and no clear instructions … It took weeks for my blood pressure to return to normal and the only thing I was told was to call if my blood pressure was too high.”*

##### Ongoing complications

Responders (93/895) indicated concerns about post-delivery complications, both immediate and long-term. More than one third of these (*n* = 31) were primarily concerned about long-term complications, such as cardiovascular disease. Some (*n* = 14) were concerned about a lack of awareness of the possible long-term risks and others (*n* = 15) wanted more detailed information about the effects of HDP on their long-term health. A few of these responders wondered whether having a pregnancy affected by HDP would increase their (*n* = 8) or their infant’s (*n* = 5) HDP risk.

*“YES!! I wish I had been told that there are sometimes long-term problems with both baby and mother! I was told nothing, except that I may develop it again in subsequent pregnancies.”*

With respect to reproductive health, 22 (out of 93) responders were worried about the impact of HDP on future pregnancies. More than half of these (*n* = 13) reported they were either told they would not develop HDP again or they were not informed about their recurrence risk. Four responders mentioned that their HDP experience shaped their future pregnancy planning.

*“My son will most likely be our only child. Our HELLP experience was so terrifying and I was so poorly managed … that we do not want to risk and have this happen again.”*

### Management and counseling

#### Request for improved treatment and communication

Nearly one quarter of all responders (213/895) expressed a desire for alternative or additional treatment than what they received. The most common request was for improved counseling from providers (*n* = 69): “*They didn’t even mention the word pre-eclampsia to me. I had no idea what was happening to me*”, “*They never explained to me what was happening*”, “*more support and better communication*”. Another challenge was that they felt providers minimized their suffering by normalizing or not taking their symptoms seriously (*n* = 22): “*Everyone told me I was either faking or ‘normal’*”, “*All [symptoms] were dismissed*”*.* Finally, others (*n* = 25) felt that their cases could have been managed better due to missed or delayed diagnoses: “*[They] should have been able to piece together my condition quicker*”, “...*it was misdiagnosed for months!!!*”

Thirty-four responders felt they received insufficient monitoring: “*I wish I had been better monitored for blood pressure*” and 36 expressed concern about test results. “*A test result was ignored – I was never informed that my protein in my urine [was] slightly elevated*”.

Some responders (*n* = 20) had specific suggestions for treatments, such as “*blood pressure medicine*” and aspirin (*n* = 7). A few were concerned about discharge care including being discharged too early (*n* = 9) and given insufficient information or follow-up after discharge (*n* = 6). Eighteen responders critiqued the timing or mode of birth. Some responders felt they should have seen a different type of provider (*n* = 11): “*I would have preferred to have been referred to maternal fetal medicine as soon as I became pregnant*”.

Regarding more immediate postpartum issues, 32 responders desired more information concerning infant support, especially for a premature infant, breastfeeding, and familiarity with the neonatal intensive care unit (NICU) care. Post-pregnancy mental health was an important issue (*n* = 48) due to concerns regarding postpartum depression and/or post-traumatic stress disorder (PTSD) (*n* = 13) and access to emotional or psychological support (*n* = 41). Responders wanted more information on “*the emotional affects [of preeclampsia] postpartum*” and “*more support*” as they “*suffered in silence”* and “*felt hopeless and very afraid*”.

*“I think postpartum counseling would have been very helpful. PTSD is a very common occurrence and I wished I had sought help earlier than I did.”*

*“I felt very alone at the NICU. A better support system … .would have been desirable.”*

#### Concerns regarding the quality of care or facility and the type of provider

Responders (200/895) mentioned the specific type of provider they were cared for by and/or the quality of care from their health care provider or facility. Forty-nine of these responders focused on the type of provider or facility: “*I’m very disappointed with the hospital I delivered at*”. Most of these comments were general and did not single out one type of provider (i.e. nurse, midwife, or physician). Although most shared experiences of poor quality of care, 22 of the responders in this group praised the care they received: “*I saw an excellent doctor*”, “*my OB was fantastic*”.

Some (*n* = 34) of these responders raised concerns about their providers’ lack of knowledge of HDPs: “*They were clueless about HELLP*”, “*Doctors and nurses need to be more informed*”, “… *if my healthcare provider had been more informed I wouldn’t have gone undiagnosed for 3 weeks and come so close to death.”*

One of the most common concerns raised about providers was that responders felt dismissed or ignored (*n* = 61): “*[providers] downplayed my disorder*”, “*I wish my doctors/practice would have listened to [my] voice*”, *“I had weekly appointments … and I don’t feel like anyone actually listened to me”*, *“[I felt] brushed aside and ignored.”* Five responders specifically reported that they were blamed by their providers for their HDP diagnosis.

### Potential action

#### Heightened symptom vigilance

Of 62 responses (out of 895) in this category, most (*n* = 47) believed they would have been more vigilant had they been aware of the potentially dangerous nature of HDP symptoms.

“*… it would have been very helpful to have been told all about the possible symptoms [ … ] so that I would have known to take it much more seriously so that I could have sought treatment earlier … those precious hours could have given them time to give me the steroid injections that my daughter needed to develop her premature lungs … ”*

A few responders (*n* = 3) attributed early diagnosis to their symptom vigilance; however, some (*n* = 10) described how despite their efforts to remain vigilant and report symptoms to their providers, circumstances led them to doubt themselves and their assessment that something was amiss. “*I wish that I had understood the significance of my symptoms … I was sent home with a prescription for indigestion when I should have been hospitalized [as I] almost died.*”

### Importance of earlier treatment

Attaining earlier treatment was a priority for 53 responders (out of 895). They recognized that the knowledge of potentially dangerous HDP symptoms either helped them receive early treatment or would have prompted them to seek it out.

*“If I had complained more, instead of thinking my symptoms were normal pregnancy symptoms, maybe my providers could have caught the preeclampsia earlier”*.

Thirty-eight responders felt that their provider should have acted earlier regarding work-up, treatment, or management, and that they had to pressure their provider or seek another provider to resolve their concerns over troubling symptoms.

*“No one even told me what preeclampsia was until 30 minutes before they started inducing me. Knowing about it in advance would have made it less traumatic and I would have sought help for my symptoms sooner.”*

When this occurred, the encouragement and support of family and friends (*n* = 7) figured prominently. *“If I wasn’t prompted by my friend to call the doctor’s office about my discomfort a week later, I would probably not be here and neither would my baby.”*

#### Need for self advocacy

Responders (76/895) indicated that given their experiences with HDPs, they wished they had advocated for themselves when interacting with providers. “*[I] wish I had been more informed about preeclampsia during my pregnancy as I would have been more proactive*.” Advocacy included topics such as understanding HDPs to improve reporting of symptoms, demanding better care, and partnering more effectively with health care providers. Most of these responders (*n* = 49) believed that their inaction was tied to a lack of information which led them to disregard or misattribute signs and symptoms.

“*I wish I knew that the level of swelling and shortness of breath I had starting in the second trimester were not … normal pregnancy symptoms. If I had complained more, instead of thinking my symptoms were normal … , maybe my providers could have caught the preeclampsia earlier.*”

Few responders in this group (*n* = 7) said they underreported symptoms out of fear of being viewed negatively by providers.

“*I did not want to complain too much … There is a large amount of pressure to be strong during pregnancy and not be ‘whiny’.*”

*“My concerns about swelling were met with jokes about being a paranoid first time mother. I wish I had been more prepared to be my own advocate...”*

Others underreported symptoms because they believed they were due to a minor cause: “*I thought I just had heartburn. I had no idea what a serious condition I was in [and] I felt silly for even calling … with chest pain*.” Most responders in this group (*n* = 46) believed that had they been better informed, they would have demanded better care, including insisting that their symptoms be taken seriously by providers. “*I wish I had known how dangerous high blood pressure was. My doctor didn’t take me serious [ly], and it took me taking my own [blood pressure] at the grocery store to realize how bad it was.*” Given the adverse outcomes of HDPs including infant loss, many responders also (*n* = 33) wrote about their hindsight for action.

*“I should have been more assertive about getting my blood pressure readings from my nurse. I would have benefitted from knowing about HELLP and how hard it is to diagnose. I wish I had gone to my doctor’s office the first day I felt sick instead of just talking to one of their nurses.”*

*“I would have liked more information [ … ], so I could have been more insistent on finding a reason [for] why I was sick.”*

Some responders (*n* = 12) claimed to have successfully advocated for themselves and a few of these responders (*n* = 4) believed they avoided adverse outcomes or long-term complications because of their knowledge of HDPs: “*I was fortunate to know enough about preeclampsia from watching pregnancy-related documentary shows on TV, that I diagnosed myself with preeclampsia … I probably would have had a seizure at home if I had not already known about preeclampsia*.” Others (n = 4), despite their lack of experience, successfully advocated for themselves by other means. One woman suggested additional tests: *“Healthcare providers were certain I had heartburn or gallstones until I had an ultrasound showing nothing wrong with the gallbladder at which point I requested bloodwork which was what led to* [*the*] *HELLP diagnosis.*” Still other responders (*n* = 2) based their persistence on “*instinct*” or “*knowing something was wrong*.”

*“When I called the hospital … the midwife didn’t feel there was any cause for alarm and told me to stay home. It was only my instinct that told me something wasn’t right. I ignored her advice and went to the hospital. That saved my life and the life of my baby. I truly believe that.”*

Others (*n* = 3) wished that they had self-advocated better: “*I wish I had been more prepared to be my own advocate and that I had been more informed of warning signs*.” Finally, a few responders (n = 2) felt that by knowing more about HDPs they could better partner with their providers: “*I wish that I was overall more informed about what my BP should be and what I should be aware of. I was so determined that if I did ‘everything right’ nothing would go wrong, so I just trusted my doctor to let me know if there was anything to worry about. I wish I had known that I should be my own advocate.*”

Prespecified subgroup analyses by year of delivery, perinatal outcome, country, and provider did not uncover any substantial differences in prominent themes (data not included).

## Discussion

By analyzing individual narrative responses of an open-ended question from a large patient registry, we report meaningful insights into the patient voice regarding HDP, a globally important obstetric complication. Although the question was designed to elicit participant needs, responders provided insights spanning the breadth of their HDP experience. We found notable thematic overlap among responses with respect to wanting additional information, desiring improved management or counseling, and preferred action by the patient and provider. Responders also indicated interest in learning about broad management topics around prevention, diagnosis, and treatment. A desire for more involvement in their care, such as being appraised of results and management plans, also stemmed from this lack of information, which led to inaction on their part or misattribution of symptoms. Additionally, responders demonstrated accountability for their care and outcomes, often indicating a desire to have better articulated their concerns to providers. Most responders explained that had they understood the severity and the rapidity with which their condition might deteriorate, they would have taken their symptoms more seriously, taken prevention steps earlier, and demanded different care. Responders’ self-directed action may be attributable to learned helplessness to affect any other type of change. The power imbalance in the provider-patient relationship may disempower patients’ ability to negotiate for their health especially in high-stakes situations when reliance on the provider as expert is increased and authentic shared decision making becomes almost impossible [20]. Similar overarching themes have been reported through smaller qualitative or survey –based studies, indicating that the perspective of the participants in our cohort may have applicability across the spectrum of clinical settings [21–24].

One period of particular concern was post-delivery, both immediate and long-term. This is a critical time in the natural history of HDP as blood pressure is often higher and eclampsia is more likely to occur postpartum [25, 26] reinforcing the need for targeted communication and postpartum monitoring. Responders also requested information regarding their long-term health, specifically cardiovascular disease, for which women with preeclampsia are at risk of [27–32]. Several also indicated poor counseling regarding the recurrence of HDP [33, 34﻿] and some were incorrectly informed that HDP would not recur. Importantly, HDP recurrence risk influenced reproductive planning for some, as they deferred future pregnancies.

Prominent throughout responses was an emphasis on patient-provider communication, highlighting the importance of communication that is transparent about the consideration of HDP as a possible diagnosis and its associated outcomes, as responders reported being unaware that they were being evaluated for this condition until the situation became more dire [24]. Sixty-five responders were unsure of or did not receive a diagnosis, a potential crude measure of ineffective communication. Several cited concerns regarding not being taken seriously by their provider, and that they were brushed aside, ignored, or blamed. This is especially disquieting given the disease severity presented in this population, suggesting a missed opportunity for HDP recognition. Responders advocated for a partnership with their providers as they recognized the challenges with HDP diagnosis: that no one test exists and that clinical scenarios rapidly change. Indeed, HDPs represent a unique pregnancy complication as they can present heterogeneously, progress rapidly, and have immediate high acuity impact on both mother and fetus, requiring complex medical decision-making. HDPs also have long-term impact on maternal and offspring health [30–32, 35﻿], adding complexity to communications. As most pregnancies proceed without incident, diagnosing HDP can be challenging, especially in nulliparas. Although a deeper understanding of the provider perspective was beyond the scope of this study, dissatisfaction in the care provided was unfortunately common, highlighting a need for improved trust in providers to improve diagnosis, care, and satisfaction [36, 37﻿].

Our study is restricted to those proficient in English and relies on participant recall, with an expected bias towards participation for those with a more severe disease presentation, limiting generalizability among those with milder forms of HDP. Although a response rate of 48.4% is not out of the ordinary given the passive nature of TPR, this may further limit generalizability. Many completed the questionnaire several years after their first HDP pregnancy (median 2.8 years) thus intervening life events, including additional pregnancies and education, may have influenced narrative responses. We anticipate, and the literature suggests, that life events that are emotionally arousing are unlikely to be forgotten, that they are accurate, and that the narrative becomes more mature the more often it is repeated [38, 39]. As some of the data was collected several years prior, it is possible that health systems, attitudes, and training may have changed in the intervening time. Most participants reported being non-Hispanic white leading to a lack of representation of other race/ethnicities. Reasons for this lack of representation in our sample are unknown, but may include access to technology, internet connectivity, or the passive nature of the registry itself, which by design is a convenience sample. Approximately 17% (310/1850) of participants in our cohort received care outside the United States and attempts to incorporate a more global perspective is warranted to better understand needs in different health care and community settings. Due to limitations of the questionnaire design at the time, it did not capture gestational hypertension or medically-indicated terminations for severe preterm HDP (now rectified). Our inclusion of the number of respondents for each subtheme and use of the terms “few”, “some”, “many”, and “most” should not discount the importance of points raised by fewer responders or unduly increase the importance of those raised by more responders. All presented results demonstrate consistency between the data presented and our findings.

Importantly, 21% participants indicated being satisfied with the information they received. As there is no standardized counseling provided to all women being evaluated for or diagnosed with preeclampsia in the US, we cannot comment on what specific information was deemed satisfactory to women, and conversely, unsatisfactory. As the US does not have a nationalized health care system, participants likely received care in diverse settings including smaller community hospitals and larger tertiary academic centers, with variable resources. Additionally, we are unable to make inferences about the HDP experiences of the more than half of all participants (955/1850) who did not provide a response to the open-ended question. We included participants who were unsure of their ultimate diagnosis (7%), potentially skewing our data if they were not truly impacted by HDP; however, medical record validation from a subset of this cohort through prior work has confirmed reliable correlation [40﻿].

## Conclusions

The impact of HDP on pregnancy, infant, and adult health outcomes is well appreciated; however, the patient’s voice in this disease experience is critically lacking. Responders in this study provide invaluable input notable for a generally poor understanding of the existence of HDP and its clinical features, along with a desire for improved communication with their providers to cultivate a partnership during their HDP experience. These considerations can inform practice changes including routine HDP education for all obstetrical patients, and for providers to actively involve patients during the often-challenging task of diagnosing HDP. Elevated patient awareness and engagement using effective patient-provider communication to promote early diagnosis and intervention, which could favorably alter the disease outcome for patients and their children.

## Data Availability

The datasets used and analyzed during the current study are available from the corresponding author on reasonable request.

## Abbreviations

HDP: Hypertensive disorders of pregnancy
TPR: The Preeclampsia Registry
HELLP: Hemolysis elevated liver enzymes low platelet count
SBP: Systolic blood pressure
DBP: Diastolic blood pressure
NICU: Neonatal intensive care unit
PTSD: Post-traumatic stress disorder
